# A comparison study of metabolic profiles, immunity, and brain gray matter volumes between patients with bipolar disorder and depressive disorder

**DOI:** 10.1186/s12974-020-1724-9

**Published:** 2020-01-30

**Authors:** Ya-Mei Bai, Mu-Hong Chen, Ju-Wei Hsu, Kai-Lin Huang, Pei-Chi Tu, Wan-Chen Chang, Tung-Ping Su, Cheng Ta Li, Wei-Chen Lin, Shih-Jen Tsai

**Affiliations:** 1 Department of Psychiatry, Taipei Veterans General Hospital, No. 201, Shih-Pai Road, Sec. 2, 11217 Taipei, Taiwan; 2 Division of Psychiatry, Faculty of Medicine, National Yang-Ming University, Taipei, Taiwan; 3 Institute of Brain Science, National Yang-Ming University, Taipei, Taiwan; 4 Department of Medical Research, Taipei Veterans General Hospital, Taipei, Taiwan; 5 Institute of Philosophy of Mind and Cognition, National Yang-Ming University, Taipei, Taiwan; 6 Department of Psychiatry, Cheng Hsin General Hospital, Taipei, Taiwan

**Keywords:** Bipolar disorder, Major depressive disorder, Pro-inflammatory cytokine, Magnetic resonance imaging, Gray matter, Voxel-based morphometry

## Abstract

**Background:**

Previous individual studies have shown the differences in inflammatory cytokines and gray matter volumes between bipolar disorder (BD) and unipolar depression (UD). However, few studies have investigated the association between pro-inflammatory cytokines and differences in brain gray matter volumes between BD and UD.

**Methods:**

In this study, 72 BD patients and 64 UD patients were enrolled, with comparable gender and age distributions (33.8% males and an average age of 39.3 ± 13.7 years). Each participant underwent metabolic profiling (including body mass index (BMI), glucose, triglyceride, high-density lipoprotein (HDL), leptin, insulin, adiponectin), pro-inflammatory cytokine (including soluble interleukin-6 receptor (sIL-6R), soluble interleukin-2 receptor (sIL-2R), C-reactive protein (CRP), soluble tumor necrosis factor receptor type 1 (sTNF-R1) examinations, and structural magnetic resonance imaging exams. Voxel-based morphometry was performed to investigate the gray matter volume differences between BD and UD patients. Correlations between pro-inflammatory cytokines and the gray matter volume difference were analyzed.

**Results:**

Compared to UD patients, the BD group had significantly higher BMI, and higher levels of sIL-6R and sTNF-R1 than the UD patients. The BMI significantly correlated with the level of pro-inflammatory cytokines. Adjusted for age, sex, BMI, duration of illness and total intracranial volume, the BD individuals had significantly more reduced gray matter volumes over 12 areas: R. cerebellar lobule VIII, R. putamen, L. putamen, R. superior frontal gyrus, L. lingual gyrus, L. precentral gyrus, R. fusiform gyrus, L. calcarine, R. precuneus, L. inferior temporal gyrus, L. hippocampus, and L. superior frontal gyrus. These 12 gray matter volume differences between BP and UD patients negatively correlated with sIL-6R and sTNF-R1 levels.

**Conclusions:**

Our results suggested that BD patients had higher BMI and pro-inflammatory cytokine levels in comparison to UD patients, especially IL-6 and sTNF-R1, which may contribute to greater gray matter reductions in BD patients in comparison to UD patients. The results support the neuro-inflammation pathophysiology mechanism in mood disorder. It is clinically important to monitor BMI, which, in this investigation, positively correlated with levels of inflammatory cytokines.

## Background

Accumulating evidence suggests that inflammatory processes play an important role in the pathogenesis, phenomenology, comorbidity, and treatment of mood disorders [1, 2]. A bidirectional circuit between the immune and neuroendocrine systems has been suggested as enabling a complex reciprocal relationship between the immune and hypothalamic-pituitary-adrenal (HPA) axis functions in unipolar depression (UD) [3]. Patients with depression show elevated peripheral inflammatory biomarkers, even in the absence of medical illness [4–6]. Patients treated with cytokines are at a greater risk of developing a depressive disorder, and the administration of anti-cytokines to patients with concurrent depression and inflammatory disease has resulted in relief of depressive symptoms [1, 7–10]. Increased expressions of inflammatory mediators in depressed patients may lead to a poor response to antidepressant drug therapy [1], affecting brain signaling patterns, cognition, and the production of a constellation of symptoms, termed “sickness behavior” [11, 12]. Regarding bipolar disorder (BD), available evidence indicates that BD and inflammation are linked through shared genetic polymorphisms and gene expressions [13], and multi-system inflammatory involvement may be present during the early stage of BD [14]. Pro-inflammatory cytokines have unique and specific actions on neurons and circuits within the central nervous system, influencing the microglial activation [15], signaling molecules in neurotransmission, memory, and glucocorticoid function, as well as activity control [16]. Inflammatory mediators may alter monoamine and glutamate neurotransmissions, glucocorticoid receptor resistance, and hippocampal neurogenesis [17].

Previous imaging studies have demonstrated that brain gray matter (GM) volume reductions in several specific regions, such as in the prefrontal cortex, occur in both BD and UD patients, and are associated with disease severity and cognitive impairment [18–21]. However, few studies have directly compared metabolic profiles, the pro-inflammatory cytokine and associated brain GM volume changes between BD and UD individuals. In general, BD is regarded as a more severe mood disorder than UD with earlier onset, more recurrent episodes, more deficits in neurocognitive function [22–25], and more pathology in neuroimaging findings with more widespread volumetric changes [26–29]. Direct comparisons of brain GM changes revealed that, compared with UD patients, BD individuals showed reduced GM volumes in the right inferior frontal gyrus, middle cingulate gyrus, hippocampus, and amygdala; indicating that BD patients exhibited a more pervasive GM volume reduction than UD patients [30, 31]. However, the mechanisms underlying the GM volume differences between BD and UD remain unknown. Our previous study found the pro-inflammatory cytokines levels of soluble interleukin-6 receptor (sIL-6R), soluble interleukin-2 receptor (sIL-2R), C-reactive protein (CRP), and soluble tumor necrosis factor receptor type 1 (sTNF-R1) were significantly higher in BD patients than in UD patients, indicating more severe inflammatory dysregulations in BD than UD [32]. Whether the GM volume reduction differences are related to a more severe inflammatory dysregulation in BD patients than UD individuals have rarely been investigated. In this study, we investigated the association between pro-inflammatory cytokines and differences in brain gray matter volumes between BD and UD. The results may contribute to the understanding of the role of inflammation dysregulation in mood disorders.

## Methods

### Participants

Patients aged between 20 and 65 years who met the Diagnostic and Statistical Manual of Mental Disorders, Fourth Edition, Text Revision (DSM-IV-TR) criteria for bipolar disorder or unipolar depression with a Clinical Global Impression-Severity (CGI-S) scale for bipolar disorder or unipolar depression ≤ 3 were included in the current study. The exclusion criteria included any DSM-IV diagnosis of the following: lifetime history of schizophrenia or any other psychosis, intellectual disability, organic mental disorder, autoimmune/immune diseases, substance abuse in the past 3 months or dependence in the past 6 months, pregnancy or breastfeeding, and unstable physical illnesses. The study was approved by the Institutional Review Board of the Taipei Veterans General Hospital and conducted in accordance with the Declaration of Helsinki. Written informed consent was obtained from all patients prior to their inclusions in the study.

### Measurements of metabolic profiles and pro-inflammatory cytokines

The metabolic profiles, including body mass index (BMI), glucose, triglyceride (TG), high-density lipoprotein (HDL), leptin, ghrelin, insulin, adiponectin, were examined. Serum glucose, triglyceride, and cholesterol levels were measured using a glucose oxidase autoanalyzer, a triglyceride enzyme autoanalyzer, and a cholesterol oxidase autoanalyzer, respectively (Dimension RxL, DADE Behring Company, Inc., Newark, DE, USA); ghrelin was measured using a radioimmunoassay (RIA) kit (Peninsula Laboratories, Inc., San Carlos, CA, USA). Insulin concentrations were analyzed using a radioimmunoassay kit (Coat-A Count Insulin; Diagnostic Product Corporation, Los Angeles, CA, USA). Serum adiponectin level was measured using a quantitative Human Adiponectin ELISA Kit (B-Bridge International, Inc., Mountain View, CA, USA). The pro-inflammatory cytokine levels, including sIL-6R, sIL-2R, CRP, and sTNF-R1, were determined using enzyme-linked immunosorbent assay (ELISA) kits (R&D Systems, Minneapolis, MN, USA). Fasting serum samples were collected in serum separator tubes, clotted for 30 min, and stored at − 80 °C until use. All assays were performed according to the vendor’s instructions. The final absorbance of each sample of the mixture was measured and analyzed at 450 nm using an ELISA plate reader with Bio-Tek Power Wave Xs and Bio-Tek’s KC junior software (Winooski, VT, USA). The standard range was considered as specified in the vendor’s instructions. A linear regression *R*^2^ value of at least 0.95 was considered a reliable standard curve.

### Magnetic resonance imaging acquisition

All brain images were acquired on a 3.0-T GE Discovery MR750 whole-body high-speed MRI device. Automated shimming procedures were performed, and scout images were obtained. A high-resolution structural image was acquired in the axial plane using the FSPGR sequence (BRAVO) on GE equipment with parameters (repetition time [TR] = 12.23 ms, echo time [TE] = 5.18 ms, inversion time [TI] = 450 ms, and flip angle = 12°) and an isotropic 1-mm voxel (FOV 256 × 256). One hundred sixty-eight contiguous horizontal 1 mm thick slices were acquired parallel to the anterior commissure-posterior commissure line. These slices covered the cerebellum of each participant. To minimize the generation of motion artifacts during image acquisition, each participant’s head was immobilized with cushions inside the coil.

### Voxel-based morphometry

Individual high-resolution T1-weighted volumetric images were processed using Statistical Parametric Mapping (SPM12, Wellcome Institute of Neurology, University College London, UK) executed in Linux-based MATLAB 2014a (MathWorks, Natick, MA, USA) with default settings. In the current study, the detailed VBM approach included the following: Data were first carefully checked by an experienced radiologist to rule out any scanner artifacts, motion problems, or gross anatomical abnormalities for each participant. After data checking and origin identification, the Segment Toolbox from SPM12 was applied to every T1-weighted MR image to extract tissue maps corresponding to gray and white matters, and cerebrospinal fluid in native space. To achieve higher accuracy of registration across subjects, all native space tissue segments were imported into a rigidly aligned space and iteratively registered to group-specific templates that were generated from all structural images in this study through nonlinear warping using the DARTEL toolbox. These images were resampled to 1.5 mm isotropic voxels. Subsequently, the resliced images of gray and white matters were registered to a subject-specific template using the DARTEL template-creation toolbox to improve inter-subject alignment, and the normalization function of the toolbox was used to normalize the individual images of gray and white matters to MNI space (1.5 mm isotropic voxel). Finally, the gray matter map of each subject was warped using their corresponding, smooth, and reversible deformation parameters to the custom template space and then to the MNI standard space. For the GM volume, the warped images of gray matter were modulated by calculating the Jacobian determinants derived from the special normalization step and by multiplying each voxel by the relative change in volume. The modulation step was performed to correct volume changes that might have occurred during nonlinear normalization. The warped modulated images of gray matter were smoothened through the convolution of an 8-mm full-width, at half-maximum isotropic Gaussian kernel before tissue volume calculation and voxel-wise group comparisons. The total intracranial volume (TIV) was determined as the sum of GM, WM, and CSF volumes [33, 34].

### Statistical analysis

To assess differences in demographic and clinical data, we used one-way analysis of variance for continuous variables and Fisher’s chi-squared test for nominal variables. *P* < 0.05 was used to indicate statistical significance. For imaging data, voxel-wise GM volume differences between the two disease groups were investigated using analysis of covariance (ANCOVA) with co-varying the age, sex, BMI, duration of illness, and TIV. To avoid possible edge effects around the margin between different tissue types, all voxels with a GM probability value < 0.2 (absolute threshold; range, 0–1) were excluded. The threshold was set at *P* < 0.05 (corrected for family-wise error rate (FEW) at the cluster level with a voxel-wise *P* < 0.001 using a combined height and extent threshold technique based on 10,000 Monte-Carlo simulations calculated through the Analysis of Functional NeuroImages (AFNI) program, 3dClustSim (the successor of AlphaSim; Cox, 1996; http://afni.nimh.nih.gov/pub/dist/doc/program_help/3dClustSim.html). In this study, the statistical threshold for each voxel was set at PFWE-corrected < 0.05, with a cluster size of at least 104 voxels as the threshold, based on the results of the Monte Carlo simulation. The results (Puncorrected < 0.001 and kE > 104) were considered statistically significant. The regional GM volumes were extracted from the significant clusters of group comparison for each participant. We analyzed the correlation between GM volume differences between the two groups and proinflammatory cytokine levels.

## Results

The demographic data of the study participants are presented in Table 1. In total, 72 patients with BD and 64 patients with UD were enrolled (33.8% males and an average age of 39.3 ± 13.7 years), with comparable gender and age distributions. The BD group had significantly higher BMI values, higher levels of sIL-6R and sTNF-R1 than the UD patients (Table 1, all *P* < 0.05). There were no significant differences in the rate of metabolic syndrome between BD and UD patients. The BMI correlated significantly with HDL (*r* = − 0.303, *P* < 0.01), leptin (*r* = 0.600, *P* < 0.01), insulin (*r* = 0.482, *P* < 0.01), adiponectin (*r* = − 0.311, *P* < 0.01), sIL-6R (*r* = 0.326, *P* < 0.01), sIL-2R (*r* = 0.250, *P* < 0.01), CRP (*r* = 0.325, *P* < 0.01), and sTNF-R1 (*r* = 0.544, *P* < 0.01) levels.

**Table 1:** Demographic data, metabolic profiles, and levels of pro-inflammatory cytokines between patients with bipolar disorder and unipolar depression

	Bipolar disorder (*n* = 72)	Unipolar depression (*n* = 64)	*P* value
Demographic data			
Sex (M/F, *n*)	27/45	19/45	0.336
Age (SD)	39.5 (12.3)	39.0 (15.3)	0.837
Metabolic profiles			
BMI (SD)	26.6 (5.2)	23.7 (4.03)	0.002*
Glucose (SD)	89.8 (16.4)	89.1 (9.2)	0.758
Triglyceride (SD)	119.5 (93.5)	107.4 (69.9)	0.400
High density lipoprotein (HDL)	56.4 (16.9)	56.8 (13.4)	0.876
Leptin (SD)	11,431.2 (10,944.5)	9013.4 (7372.7)	0.159
Insulin (SD)	9.65 (14.78)	8.12 (14.04)	0.551
Adiponectin (SD)	6158.4 (4670.2)	7505.1 (5664.9)	0.141
Metabolic syndrome (%)	23.6%	22.6%	0.527
Inflammation index (pg/ml)			
sIL-6R (SD)	36,917.86 (11,001.38)	29,420.91 (8282.58)	< 0.001**
sIL-2R (SD)	747.39 (323.22)	671.83 (246.70)	0.135
CRP (SD)	1751.60 (1929.26)	1823.09 (2289.56)	0.840
sTNFR1 (SD)	1216.55 (468.646)	748.46 (161.44)	< 0.001**

*BMI* body mass index, *SD* standard deviation, *MARDS* Montgomery-Åsberg Depression Rating Scale, *YAMRS* The Young Mania Rating Scale; Global Assessment of Function Scale, *sIL-2R* soluble IL-2 receptor, *sIL-6R* soluble IL-6 receptor, *CRP* C-reactive protein, *sTNFR1* soluble tumor necrosis factor-α receptor-1

*P < 0.05, **P < 0.001

Among the 64 patients with UD, there were 19 (29.7%) patients with selective serotonin reuptake inhibitors (SSRIs), 18 (28.1%) patients with serotonin-norepinephrine reuptake inhibitors (SNRIs), 11 (17.2%) patients with norepinephrine dopamine reuptake inhibitors (NDRIs), 4 (6.3%) patients with noradrenergic and specific serotonergic antidepressants (NaSSAs), and 12 (18.7%) with agomelatine. Among the 72 patients with BD, 11 (15.3%) patients were treated with lithium or valproic acid only, 18 (25%) patients were treated with atypical antipsychotics only, 36 (50%) patients were treated with lithium or valproic acid plus atypical antipsychotics, and 7 (9.7%) patients were treated with other medications including lamotrigine and carbamazepine. To investigate the influence of medications on cytokine levels, ANOVA tests were performed, and no significant differences in any of the cytokines were noted among patients taking different groups of medications in the BD or UD group (Tables 2 and 3).

**Table 2:** Comparison of cytokines among patients with unipolar depression taking different types of antidepressant

	SSRI (*n* = 19)	SNRI (*n* = 18)	NDRI (*n* = 11)	NaSSA (*n* = 4)	Agomelatine (*n* = 9)	Significance
C-reactive protein (CRP) (pg/ml)	2322.6 ± 3028.6	999.8 ± 1408.8	3133.2 ± 2206.4	978.3 ± 605.4	1366.4 ± 1930.0	n.s.
Soluble interleukin-2 receptor (sIL-2R) (pg/ml)	643.7 ± 211.2	611.5 ± 177.7	851.0 ± 358.3	825.1 ± 316.8	561.7 ± 220.8	n.s.
Soluble interleukin-6 receptor (sIL-6R) (pg/ml)	29,504.2 ± 5970.7	28,075.4 ± 9047.5	29,518.2 ± 9497.3	35,502.9 ± 8097.8	28,205.8 ± 11,328.8	n.s.
Soluble tumor necrosis factor receptor type 1 (sTNF-R1) (pg/ml)	774.7 ± 134.7	753.1 ± 147.2	779.0 ± 235.0	863.9 ± 583.3	653.0 ± 181.7	n.s.

*NaSSA* noradrenergic and specific serotonergic antidepressant, *NDRI* norepinephrine dopamine reuptake inhibitor, *SNRI* serotonin-norepinephrine reuptake inhibitor, *SSRI* selective serotonin reuptake inhibitor, *n.s.* not significant

**Table 3:** Comparison of cytokines in patients with bipolar disorder taking a different type of treatment

	Li or VPA only (*n* = 11)	AA only (*n* = 18)	Li or VPA plus AA (*n* = 36)	Lamotrigine or carbamazepine (*n* = 7)	Significance
C-reactive protein (CRP) (pg/ml)	1725.3 ± 1053.7	1888.2 ± 2337.9	1701.7 ± 1449.0	1088.2 ± 1085.4	n.s.
Soluble interleukin-2 receptor (sIL-2R) (pg/ml)	692.3 ± 266.6	797.9 ± 337.5	760.2 ± 352.2	630.5 ± 250.1	n.s.
Soluble interleukin-6 receptor (sIL-6R) (pg/ml)	37,253.7 ± 6243.3	36,311.6 ± 10,217.6	38,994.4 ± 12,634.2	29,989.6 ± 8728.0	n.s.
Soluble tumor necrosis factor receptor type 1 (sTNF-R1) (pg/ml)	1203.3 ± 311.3	1050.6 ± 319.1	1319.2 ± 577.5	1158.1 ± 200.1	n.s.

*AA* atypical antipsychotic, *VPA* valproic acid, *n.s.* not significant

**Fig. 1:**
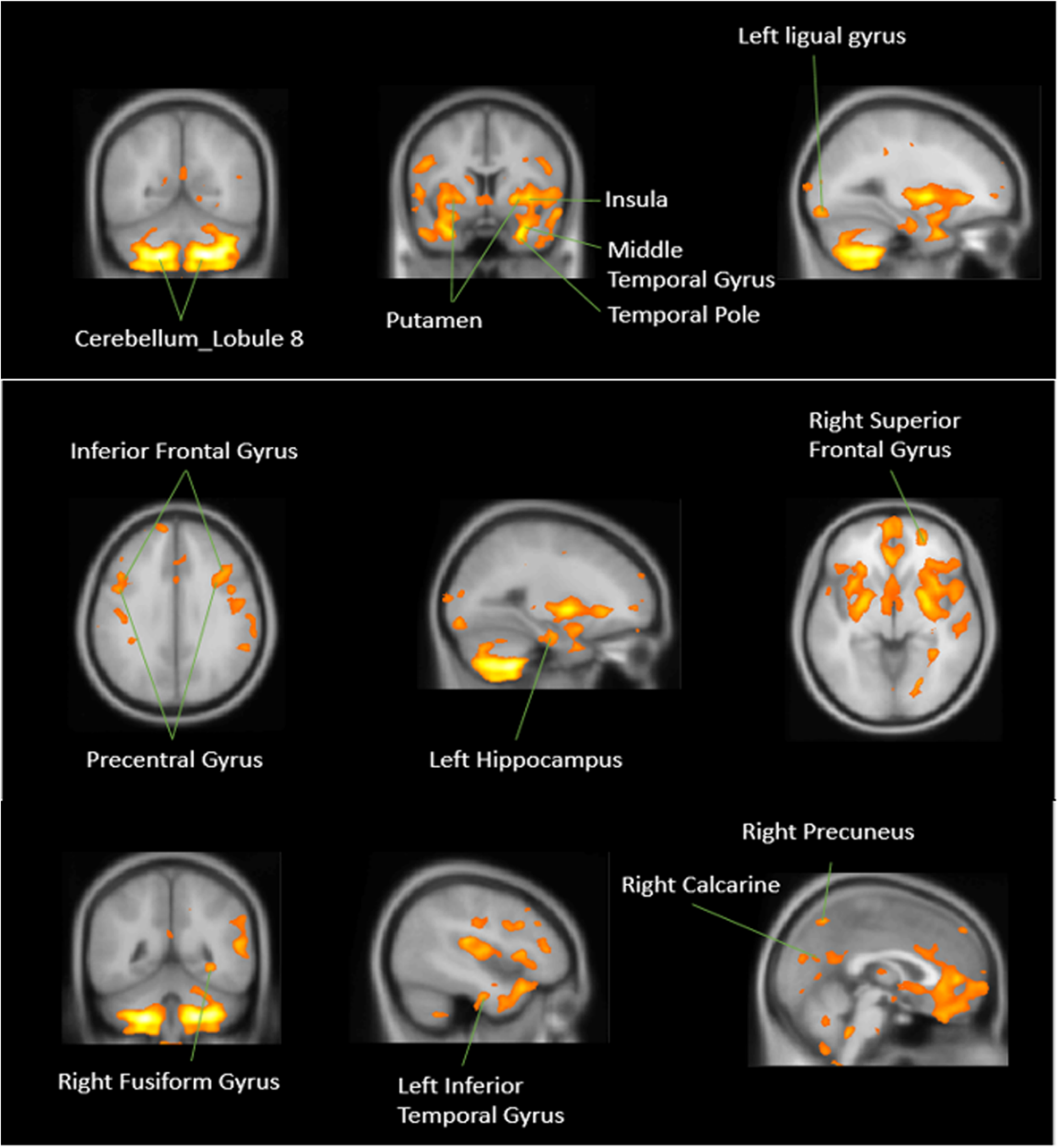
Gray matter volume differences between bipolar disorder (BD) and unipolar depression (UD) (UD > BD)^a^. **a** Adjusted for age, sex, BMI, duration of illness, and total intracranial volume (TIV)

For the comparison of gray matter, none of the brain regions were larger in patients with bipolar disorder than they were in patients with unipolar depression. BD patients had significantly reduced gray matter volume over 12 areas: R. cerebellar lobule VIII, R. putamen, L. putamen, R. superior frontal gyrus, L. lingual gyrus, L. precentral gyrus, R. fusiform gyrus, L. calcarine, R. precuneus, L. inferior temporal gyrus, L. hippocampus, L. superior frontal gyrus, adjusted for age, sex, BMI, duration of illness, and TIV (Table 4, Fig. 1). These 12 gray matter volume differences between BP and UD negatively correlated with sIL-6R, sTNF-R1 levels (Table 5).

**Table 4:** Gray matter volume differences between bipolar disorder (BD) and unipolar depression (UD) ^a^

Index	Harvard-Oxford Cortical Structural Atlas	*x*	*y*	*z*	Cluster size	*T* value	*P* value (FDR corr.)
BD > UD							
		–	–	–	–	–	–
UD > BD							
1	R. cerebellar lobule VIII	20	− 58	− 50	15,271	8.15	< 0.001**
2	R. putamen	29	− 3	− 2	3835	5.92	< 0.001**
3	L. putamen	− 26	− 3	− 1	3437	5.82	< 0.001**
4	R. superior frontal gyrus	25	53	− 5	801	4.43	0.001*
5	Left lingual gyrus	− 27	− 91	− 13	679	4.34	0.001*
6	L. precentral gyrus	− 48	6	29	543	4.26	0.001*
7	R. fusiform gyrus	32	− 48	− 6	290	4.25	0.001*
8	L. calcarine	− 13	− 77	7	216	4.19	0.001*
9	R. precuneus	1	− 63	53	258	4.19	0.001*
10	L. inferior temporal gyrus	− 44	− 14	− 31	355	4.13	0.001*
11	L. hippocampus	− 25	− 14	− 23	143	4.09	0.001*
12	L. superior frontal gyrus	− 17	56	− 12	238	3.93	0.002*

^a^Adjusted for age, sex, BMI, duration of illness, and total intracranial volume (TIV)

**Table 5:** Correlation between gray matter volume reduction and levels of pro-inflammatory cytokines

Region	R. cerebellar lobule VIII	R. putamen	L. putamen	R. superior frontal gyrus	L. lingual gyrus	L. precentral gyrus	R. fusiform gyrus	L. calcarine	R. precuneus	L. inferior temporal gyrus	L. hippocampus	L. superior frontal gyrus
sTNFR1	− 0.324**	− 0.352**	− 0.360**	− 0.217*	− 0.196*	− 0.289**	− 0.335**	− 0.289**	− 0.195*	− 0.423**	−0.168	− 0.138
sIL-6R	− 0.173**	− 0.321**	− 0.355**	− 0.254**	− 0.181*	− 0.284**	− 0.188*	− 0.212*	− 0.176*	− 0.232**	− 0.099	− 0.194**
sIL-2R	− 0.048	− 0.109	− 0.103	− 0.028	− 0.013	− 0.098	− 0.104	− 0.120	− 0.047	− 0.121	0.007	0.003
CRP	0.007	− 0.054	− 0.033	− 0.058	− 0.010	− 0.006	0.019	− 0.038	0.117	− 0.085	− 0.110	0.007

*R* right, *L* left, *sIL-2R* soluble IL-2 receptor, *sIL-6R* soluble IL-6 receptor, *CRP* C-reactive protein, *sTNFR1* soluble tumor necrosis factor-α receptor-1

Italicized data indicate the statistical significance

**p < 0.01, *p < 0.05

## Discussion

In this study, we found that BD patients had significantly higher levels of sIL-6R, sTNF-R1 levels than the UD patients. Our first study with different sample of 109 patients with UD, has found that the level of pro-inflammatory cytokines correlated with the severity of depressive symptoms [35]. Then, we enrolled other 130 BD patients, and 149 UD patients, we found the BD patients had significantly higher levels of cytokines than UD patients [30]. Among the 130 BD patients, we further found the patients with bipolar I disorder had significantly higher levels of sTNF-R1 than the patients with bipolar II disorder; the patients in manic/hypomanic states had significantly higher levels of sTNF-R1than the patients in a depressive state [36]. Combined with our previous [30, 35, 36] and the present studies with different samples, our series reports supported the pro-inflammatory cytokines may be a potential biomarker for mood disorders, and BD patients had higher immune dysregulations than UD patients.

In this study, we further investigated the association between brain pro-inflammatory cytokines and GM volume changes between BD and UD patients. We found that the BD group had significantly reduced GM volumes over 12 areas: R. cerebellar lobule VIII, R. putamen, L. putamen, R. superior frontal gyrus, L. lingual gyrus, L. precentral gyrus, R. fusiform gyrus, L. calcarine, R. precuneus, L. inferior temporal gyrus, L. hippocampus, L. superior frontal gyrus, adjusted for age, sex, BMI, duration of illness, and total intracranial volume. Furthermore, these 12 GM volume differences between BP and UD patients negatively correlated with sIL-6R, sTNF-R1 levels. These results supported our study hypothesis that BD patients have higher levels of pro-inflammatory cytokines, which associated with greater widespread GM volume changes. The meta-analysis suggested that MDD and BD are characterized by common patterns of gray-matter volume changes [37]. Our results may offer evidence that cytokine can be a biomarker for gray-matter volume change in mood disorders. As far as the differences in brain volumes preferentially in the left or right sides for some areas in our study, we did not check the patients preferentially handed. Ocklenburg et al. indicated that the structural brain correlates of handedness are unlikely to be rooted in macroscopic gray matter area differences that can be assessed with VBM [38]. In fact, most clinical imaging studies showed different left or right side brain areas in results. Few studies can have consistent bilateral symmetrical findings. These may be related to sample size and inter-individual lateralization differences.

Our results showed that IL-6, sTNF-R1 in particular may contribute to greater GM reductions in bipolar disorder in comparison to UD patients. There were some studies which supported our findings. sIL-6R has been consistently observed to be higher in patients with BD [32, 36, 39, 40]. Another study also showed sIL-6 R level reflecting the illness activity in bipolar disorder [41]. In a 13-year longitudinal study, higher levels of systemic inflammatory marker IL-6 in childhood were associated with hypomanic symptoms in young adulthood [42]. Higher sIL-6R levels were also associated with lower functional connectivity between the medial prefrontal cortex (mPFC) and subcortical structures involved in emotional processing in BD patients [43]. In patients with UD, increased IL-6 levels were associated with decreased performance on simple and choice movement time tasks [44]. For healthy subjects, previous studies also demonstrated an association between subgenual cingulate activity and mesolimbic connectivity with elevated IL-6 [45]. Among 1841 participants aged 65 to 80 community-dwelling elderly free of dementia, higher IL-6 levels were associated with lower gray matter and hippocampal volumes, and increased CSF volumes in a dose-relationship pattern [46]. Other studies also showed that IL-6 was associated with decreased total brain volume [47], hippocampal gray matter volume [48, 49], cortical atrophy [50], increased white matter hyperintensities [44], and also the rate of cortical thinning during the aging process [51]. The higher mean IL-6 concentrations were associated with accelerated annual rates of cortical thinning in the inferior temporal poles, transverse frontopolar gyri within the left hemisphere, and subcentral gyrus and sulcus within the right hemisphere [52]. Regarding sTNF-R1, a higher level of sTNF-R1 is associated with general disease severity, psychotic features and deteriorated function among patients with bipolar disorder and schizophrenic patients [52]. TNF-alpha activates an apoptotic signaling cascade leading to apoptosis and cell death, and also acts through other signaling networks impacting neuronal development, synaptic transmission, and cell survival [53]. TNF-alpha is also associated with endothelial leakage and endothelial cell activation [54], neurotoxicity, and neuroplasticity [55], and is associated with a generally negative effect on emotions and cognition [56]. Recent studies also implicated a role for TNF-alpha in neurotransmission and other aspects of neuronal function [57], and interaction with both dopaminergic and serotonergic systems [58]. The TNF-alpha level was found to have a negative correlation with cognitive function in bipolar disorder [59] and was associated with impaired executive functioning in inhibitory control and motor programming among bipolar patients [60]. Furthermore, the level of TNF-alpha was reportedly suggested to be the response predictor of lithium [61]. Our present study showed 12 gray matter volume differences between BP and UD patients negatively correlating with sIL-6R and sTNF-R1 levels. The 12 brain areas covered most findings in previous studies, including hippocampal gray matter volume [48], the inferior temporal poles, left frontopolar gyri, and right subcentral gyrus [51]. In summary, our study results suggest that elevated immune-inflammatory signaling is relevant to the pathophysiology of mood disorders.

In this study, we also found that the BD patients had significantly higher BMI than UD patients. The results were consistent with our previous 10-year cohort study that BP patients have increased risks of metabolic abnormalities in comparison with the UD patients [62]. We further found that BMI significantly correlated with the level of proinflammatory cytokines. Based on the results showing the reduced gray matter volume associated with the level of inflammatory cytokines, it is clinically important to monitor BMI. The previous study showed that patients with metabolic syndrome had significant reductions in mean cortical thickness and volume in both hemispheres compared with controls [60]. Our previous study also found that BD patients with obesity and metabolic diseases are associated with poor clinical outcomes, including more hospitalizations, more severe tardive dyskinesia, poor insight, poor global function, and more impaired executive function [63]. Other studies also found that BD patients with obesity had a longer illness duration, poorer global function, more disabilities, and poorer response to lithium [64], and poor cognitive function [65–68] than non-obese patients did.

There are some limitations to our study. First, our study is a cross-sectional study design and the patients were not drug-free. In addition to the variables adjusted for in the regression model (i.e., age, gender, illness duration, BMI and ITV), psychiatric medication, such as mood stabilizers and atypical antipsychotics, are known to cause metabolic adverse effects, inflammatory cytokines, and brain gray matter changes. Allowing patients to continue their medications was ethically more appropriate and prevented disease relapse; also, it could provide more naturalistic data. In our analysis, similar to our previous reports [30], no significant differences in any of the cytokines were noted among patients taking different groups of medications in the BD or UD group, but the effects of medication on cytokines are still difficult to elucidate. A drug-free and prospective study design may be required to confirm our findings. Second, we enrolled a group of patients who were in a mildly ill condition (CGI-S ≤ 3), which included remitted, hypomanic or minor depression states, in the current study. The metabolic, immune, and brain gray matter changes in the acute phase of bipolar disorder and unipolar depression and in different mood states would need further investigation. Third, the inflammatory cytokines can be influenced by body weight and medical comorbidity, and the subjects without bipolar or unipolar disorder may still have high cytokine levels under the circumstances. Therefore, the high cytokine level alone is not a single factor to differentiate mood disorders, and the associated factors should be considered. Finally, there was no healthy control group in the present study. Future studies with a control group are required to validate the results.

## Conclusions

Our results suggested that BD patients had higher BMI and pro-inflammatory cytokines levels than UD patients, especially IL-6 and sTNF-R1, which may contribute to greater gray matter reductions in BD patients in comparison to UD patients. The results support the neuro-inflammation pathophysiology mechanism in mood disorder. It is clinically important to monitor BMI, which positively correlated with levels of inflammatory cytokines.

## Data Availability

The datasets generated during and/or analyzed during the current study are not publicly available due to the intelligence rights owned by the hospital and the authors but are available from the corresponding author on reasonable request.

## Abbreviations

AFNI: The Analysis of Functional NeuroImages
ANCOVA: Analysis of covariance
BD: Bipolar disorder
BMI: Body mass index
CGI-S: Clinical Global Impression-Severity
CRP: C-reactive protein
DSM-IV-TR: The Diagnostic and Statistical Manual of Mental Disorders, Fourth Edition, Text Revision
ELISA: Enzyme-linked immunosorbent assay
FEW: Family-wise error rate
GM: Gray matter
HDL: High-density lipoprotein
HPA: Hypothalamic-pituitary-adrenal
MetS: Metabolic syndrome
mPFC: Medial prefrontal cortex
sIL-2R: Soluble interleukin-2 receptor
sIL-6R: Soluble interleukin-6 receptor
sTNF-R1: Soluble tumor necrosis factor receptor type 1
TIV: Total intracranial volume
UD: Unipolar depression
